# Time management and academic achievement among medical students: a cross-sectional study

**DOI:** 10.1186/s12909-026-08813-8

**Published:** 2026-02-18

**Authors:** Methaq H Alogaili, Zeena A Hussein, Afnan A Alsallami, Sadeq H. Khaleel

**Affiliations:** 1 Department of family and community medicine, College of Medicine, Al-Nahrain University, Baghdad, Iraq; 2 Department of pharmacology, College of Medicine, Al-Nahrain University, Baghdad, Iraq; 3 College of Medicine, Al-Nahrain University, Baghdad, Iraq

**Keywords:** Medical education, Time management, Academic achievement, Medical students, GPA

## Abstract

**Background:**

Time management involves using time efficiently to complete specific tasks and the grade point average (GPA) is a summary statistic that represents students’ average academic performance over a specific period. Both the effective and wise use of time are key factors influencing the productivity and success of medical students. To the best of our knowledge, no studies in Iraq have addressed this topic.

**Objectives:**

This study aims to examine the impact of time management on the academic GPA performance of students at Al-Nahrain College of Medicine and to explore their time management practices.

**Methods:**

This cross-sectional study was conducted from June 2024 to May 2025 with a total of 382 male and female students. Data was collected from participants following a direct interview using a specially designed questionnaire and the GPAs for the students were extracted.

**Results:**

Of the 382 participants, 50.3% were female. Most participants (45.8%) had a GPA between 2.00 and 3.00. There was no significant association between student perception and GPA. The results showed a significant association between GPA and a student’s preference for managing their time daily (p value 0.01). There was a significant association between GPA and laziness, study mood when sleep pattern is insufficient (p value 0.01).

**Conclusions:**

Daily time management and adequate sleep were significantly associated with higher GPAs, while students’ perceptions showed no association with academic achievement. Focused students’ education and comprehensive strategies to enhance effective time management skills among them are recommended.

## Introduction

Although the definition for the term “time management” varies considerably, all the definitions are describing the same concept; the effective use of time in order to accomplish certain activities aiming at a goal or productivity [1, 2]. According to Oxford English Dictionary (OED), the earliest use of this term goes back to 1910s [3]. All studies conducted in this context concluded that time management is important for academic performance and achievement [4–7]. It seems that the relationship between time management and academic success is a positive relationship, as while time management leads to improvement at the academic level, poor handling of time and procrastination in assignments results in a decline at the academic level and subsequent feelings of dissatisfaction, demotivation, stress and sometimes even depression negatively affecting the ability to build time schedules and handling future tasks [8]. It is proposed that, in simple terms, time scheduling for students consists of a balance between studying hours and hours for other various productive and non-productive activities outside the studying scope throughout a 24 h daily window with relaxing and free time included [9]. Effectiveness by definition is a measure of productivity by time factor [10]. Psychological aspects like mood and overall sense of well-being that influence mental health considerably affect the ability to perform tasks in a time-Table [11]. A grade point average (GPA) is a summary statistic that represents a student’s average academic performance over a specific period of time, such as a single semester. Because it is a numerical measure, the GPA is usually calculated to two decimals. It is used to measure a student’s performance levels against established standards and to rank groups of students [12].

Studying medicine is considered difficult and requires sufficient time and effort, in addition to being considered somewhat stressful for students. The main factor influencing the productivity and success of medical students is efficient and wise utilization of time [13]. The main purpose of this study is to address this pivotal point in medical students’ academic life and its subsequent effect on their college grades. Up to our knowledge, there are no studies in Iraq addressing this issue; furthermore, few studies around the globe examined the effect of time management on medical students.

### Objectives

This study aims to demonstrate the impact of time arrangement on academic achievement of Al-Nahrain medical students and to explore time practice among them.

## Methods

A cross-sectional study was done for the period from June 2024 to May 2025. A total of 382 students (calculated using single proportion formula for finite population) from the second (130), third (126) and fourth (126) stages at Al-Nahrain University, College of Medicine, participated in the study. Students in their first, fifth and sixth academic stage where excluded from the study. Data was collected through direct interview using a validated questionnaire [14], which includes the student stage in addition to questions related to time management (a total of 22 questions) each question included five answers (strongly disagree, disagree, neutral, agree and strongly agree) and the GPAs for the students were extracted.

### Statistical analysis

Data was entered and analyzed using SPSS version 26. Likert scale scores were expressed using numbers of responses and frequencies. Chi square test was used to examine the association between student perception and behavior for time management with students’ GPA. P value less than 0.05 was considered statistically significant.

## Results

**Fig. 1:**
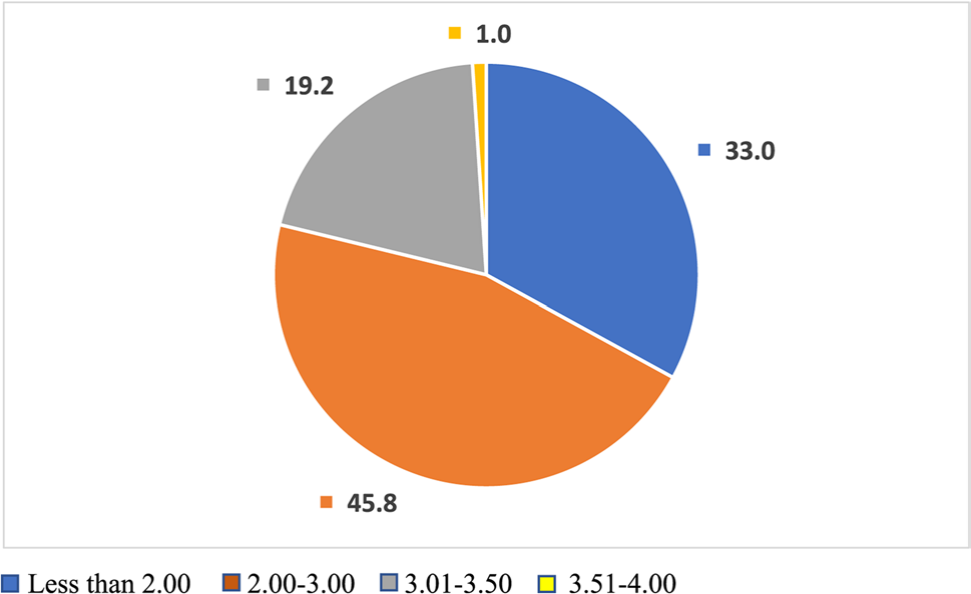
Percentage of students with various GPAs

Among 382 participants, (50.3%) of them were female, (34%) in second stage, while third and fourth stage were (33%) for each. Most of the participants (45.8%) achieved GPA between (2.00–3.00) while only (1%) achieved GPA between (3.51-4.00) (Fig. 1).

Regarding students’ perception (27.5%) of students disagree with the statement of no effect of preplanning on academic performance (Table 1).

**Table 1:** Frequency of the response related to time management on likert scale

Questions	*N* (%)				
	Strongly disagree	disagree	Neutral	Agree	Strongly agree
Student perception					
1.Preplanning methods are inefficient, and it does not affect academic performance	91(23.8)	105(27.5%)	83(21.7%)	56(14.7%)	47(12.3%)
3. My academic performance is degraded due to mis-planning	19(5.0%)	55(14.4%)	77(20.2%)	126(33.0%)	105(27.5%)
20. My participating in extracurricular activities won’t affect my academic performance.	30(7.9%)	97(25.4%)	132(34.6%)	92(24.1%)	31(8.1%)
Positive Attitude					
4. I prefer to manage my time daily.	17(4.5%)	71(18.6%)	80(20.9%)	101(26.4%)	113(29.6%)
12. I meet the deadline for any work.	26(6.8%)	72(18.8%)	131(34.3%)	92(24.1%)	61(16.0%)
14. I effectively manage workload.	24(6.3%)	75(19.6%)	119(31.2%)	118(30.9%)	46(12.0%)
19. I can adapt and be flexible when changes occur, reassess priorities, and still produce quality work.	21(5.5%)	62(16.2%)	126(33.0%)	132(34.6%)	41(10.7%)
21. I balance between my private time and study time.	53(13.9%)	107(28.0%)	107(28.0%)	76(19.9%)	39(10.2%)
16. I priorities between various competing tasks.	11(2.9%)	30(7.9%)	127(33.2%)	135(35.3%)	79(20.7%)
Negative attitude					
13. I often tend to delay/postpone my tasks.	13(3.4%)	65(17.0%)	105(27.5%)	117(30.6%)	82(21.5%)
Factor with negative impact					
2. I manage stress when handling multiple conflicting duties.	19(5.0%)	63(16.5%)	117(30.6%)	117(30.6%)	66(17.3%)
10. I have enough time to complete my tasks during the day	91(23.8%)	119(31.2%)	80(20.9%)	60(15.7%)	32(8.4%)
11. I usually understand the whole lectures.	35(9.2%)	81(21.2%)	122(31.9%)	97(25.4%)	47(12.3%)
Short time management					
5. I have a clear established plan for each week’s tasks.	45(11.8%)	103(27.0%)	99(25.9%)	87(22.8%)	48(12.6%)
6. I have a clear established plan for each month’s tasks.	53(13.9%)	120(31.4%)	107(28.0%)	62(16.2%)	40(10.5%)

There was no significant association between students’ response and GPA (p value 0.74). All statements of student perception showed no significant association between students’ perception and GPA (Table 2.).

**Table 2:** The association between student perception and GPA

Student Perception						
Questions	**Answers**	**GPA**				***P*** ** value**
		Less than 2.00	2.00–3.00	3.01–3.50	3.51-4.00	
1. Preplanning methods are inefficient, and it does not affect academic performance.	Strongly disagree	25(27.5%)	44(48.4%)	20(22%)	2(2.2%)	0.74
	disagree	34(32.4%)	49(46.7%)	22(21%)	0(0%)	
	Neutral	31(37.3%)	36(43.4%)	14(16.9%)	2(2.4%)	
	Agree	17(30.4%)	26(46.4%)	13(23.2%)	0(0%)	
	Strongly agree	19(40.4%)	20(42.6%)	8(17%)	0(0%)	
3. My academic performance is degraded due to mis-planning	Strongly disagree	6(31.6%)	10(52.6%)	3(15.8%)	0(0.0%)	0.99
	disagree	18(32.7%)	22(40.0%)	14(25.5%)	1(1.8%)	
	Neutral	23(29.9%)	37(48.1%)	16(20.8%)	1(1.3%)	
	Agree	41(32.5%)	61(48.4%)	23(18.3%)	1(0.8%)	
	Strongly agree	38(36.2%)	45(42.9%)	21(20.0%)	1(1.0%)	
20. My participating in extracurricular activities won’t affect my academic performance.	Strongly disagree	11(36.7%)	15(50.0%)	3(10.0%)	1(3.3%)	0.15
	disagree	31(32.0%)	39(40.2%)	27(27.8%)	0(0.0%)	
	Neutral	45(34.1%)	64(48.5%)	21(15.9%)	2(1.5%)	
	Agree	25(27.2%)	48(52.2%)	19(20.7%)	0(0.0%)	
	Strongly agree	14(45.2%)	9(29.0%)	7(22.6%)	1(3.2%)	

*P* value less than 0.05 considered significant

Regarding positive and negative attitudes, results show significant association between GPA and students’ preference of managing their time daily (p value 0.01). While it shows no significance association (p value 0.06) between GPA and students who can adapt and be flexible when changes occur in their plans (Table 3).

**Table 3:** The association between behavior for time and GPA of last year

Behavior for time management	Answers	GPA				*P* value
		Less than 2.00	2.00–3.00	3.01–3.50	3.51-4.00	
Positive Attitude						
4. I prefer to manage my time daily	Strongly disagree	8(47.1%)	6(35.3%)	3(17.6%)	0(0.0%)	**0.01**
	disagree	31(43.7%)	29(40.8%)	11(15.5%)	0(0.0%)	
	Neutral	36(45.0%)	33(41.3%)	9(11.3%)	2(2.5%)	
	Agree	28(27.7%)	45(44.6%)	27(26.7%)	1(1.0%)	
	Strongly agree	23(20.4%)	62(54.9%)	27(23.9%)	1(0.9%)	
12. I meet the deadline for any work.	Strongly disagree	10(38.5%)	10(38.5%)	5(19.2%)	1(3.8%)	0.56
	disagree	24(33.3%)	37(51.4%)	11(15.3%)	0(0.0%)	
	Neutral	38(29.0%)	64(48.9%)	27(20.6%)	2(1.5%)	
	Agree	36(39.1%)	34(37.0%)	22(23.9%)	0(0.0%)	
	Strongly agree	18(29.5%)	30(49.2%)	12(19.7%)	1(1.6%)	
14. I effectively manage workload.	Strongly disagree	5(20.8%)	15(62.5%)	4(16.7%)	0(0.0%)	0.27
	disagree	35(46.7%)	28(37.3%)	12(16.0%)	0(0.0%)	
	Neutral	35(29.4%)	56(47.1%)	27(22.7%)	1(0.8%)	
	Agree	33(28.0%)	59(50.0%)	24(20.3%)	2(1.7%)	
	Strongly agree	18(39.1%)	17(37.0%)	10(21.7%)	1(2.2%)	
19. I can adapt and be flexible when changes occur, reassess priorities, and still produce quality work.	Strongly disagree	12(57.1%)	7(33.3%)	2(9.5%)	0(0.0%)	0.06
	disagree	23(37.1%)	29(46.8%)	9(14.5%)	1(1.6%)	
	Neutral	40(31.7%)	68(54.0%)	18(14.3%)	0(0.0%)	
	Agree	38(28.8%)	55(41.7%)	37(28.0%)	2(1.5%)	
	Strongly agree	13(31.7%)	16(39.0%)	11(26.8%)	1(2.4%)	
21. I balance between my private time and study time.	Strongly disagree	24(45.3%)	23(43.4%)	6(11.3%)	0(0.0%)	0.17
	disagree	41(38.3%)	45(42.1%)	19(17.8%)	2(1.9%)	
	Neutral	29(27.1%)	56(52.3%)	22(20.6%)	0(0.0%)	
	Agree	21(27.6%)	35(46.1%)	18(23.7%)	2(2.6%)	
	Strongly agree	11(28.2%)	16(41.0%)	12(30.8%)	0(0.0%)	
16. I priorities between various competing tasks.	Strongly disagree	6(54.5%)	4(36.4%)	1(9.1%)	0(0.0%)	0.14
	disagree	13(43.3%)	13(43.3%)	4(13.3%)	0(0.0%)	
	Neutral	47(37.0%)	58(45.7%)	19(15.0%)	3(2.4%)	
	Agree	33(24.4%)	65(48.1%)	37(27.4%)	0(0.0%)	
	Strongly agree	27(34.2%)	35(44.3%)	16(20.3%)	1(1.3%)	
Negative attitude						
13. I often tend to delay/postpone my tasks	Strongly disagree	6(46.2%)	3(23.1%)	4(30.8%)	0(0.0%)	0.13
	disagree	16(24.6%)	35(53.8%)	12(18.5%)	2(3.1%)	
	Neutral	34(32.4%)	49(46.7%)	21(20.0%)	1(1.0%)	
	Agree	32(27.4%)	56(47.9%)	28(23.9%)	1(0.9%)	
	Strongly agree	38(46.3%)	32(39.0%)	12(14.6%)	0(0.0%)	

*P* value less than 0.05 considered significant

Regarding factors with negative impact, there was a significant association between GPA and laziness, study mood when sleep pattern is insufficient (p value 0.01) (Table 4).

**Table 4:** Relationship between various factors of time management and GPA

Questions	Answers	GPA				*P* value
		Less than 2.00	2.00–3.00	3.01–3.50	3.51-4.00	
Factors with negative impact						
9. I feel lazy and my study mood is ruined because of insufficient sleeping-pattern.	Strongly disagree	12(54.5%)	8(36.4%)	2(9.1%)	0(0.0%)	**0.01**
	disagree	17(37.0%)	17(37.0%)	11(23.9%)	1(2.2%)	
	Neutral	23(31.9%)	31(43.1%)	15(20.8%)	3(4.2%)	
	Agree	40(40.8%)	42(42.9%)	16(16.3%)	0(0.0%)	
	Strongly agree	34(23.6%)	77(53.5%)	33(22.9%)	0(0.0%)	

*P* value less than 0.05 considered significant

## Discussion

In the 21st century, we are living in a time of speed. Every task seems to be under a restricted time table even the personal aspects like meals, sports are also under the pressure of a designed start and endpoint. Medical students especially are faced with the need of managing their time and meeting a desired GPA while still being able to socialize and engage in different activities outside the scope of studying. In this study, half of the participants were females, nearly half of the participants fell under an average GPA (2.00–3.00), while the other half shared the rest of GPA rankings. Unexpectedly, only 1% of medical students that participated in the study achieved a high GPA (above 3.5), data from the Association of American Medical College recorded an average GPA of 3.71 in the 2023–2024 academic year, furthermore, two-thirds of applicants with a GPA above 3.79 are accepted to medical school [15]. This huge difference from our study maybe explained by the significant discrepancy between the qualifications and academic levels of school students accepted into Iraqi’s medical colleges and the actually required qualification to achieve high GPA in these medical colleges.

As shown in Table 1, a large portion of participants agreed on the importance of pre-planning, opposing the idea that it is ineffective and does not interfere with academic performance. Again, more than half of study participants agreed with the negative influence of mis-planning on their academic performance. Planning is the most important and most effective element alongside time management, organization of possible time utilization ahead of starting any academic task will save a student from postponing, delaying other tasks or do it on the expense of personal time highlighting the students’ perception of a great impact of pre-planning on academic performance regardless their GPA [16]. While a study done in Egypt, found a statistically significant association between medical student’s perception and GPA, a larger sample size (747), explains the difference to our study results [17].

Students’ perception about the effect of participating in extracurricular activities was neutral, although nearly half (41.9%) disagreed with their ability to balance private time and study time. Again, not associated with students’ GPA. Students’ perception is a reflection of their mental capability to build a time table and follow time management practices although this is not always associated with adapting ideas into a daily practice sometimes because of reasons related to poor knowledge about time management practices or effective learning strategies as the latter was found in a study done in Saudi Arabia to has a statistically significant association with academic achievement [18].

Most of the participating students preferred to manage their time on a daily basis and this practice was strongly associated with higher GPA as shown in Table 3. The article, by Adams and Blair, 2019 mentioned an association between daily time management and better academic achievement, attributing it to a sense of “control” [19]. Simons and Galotti, 1992 studied daily time management among 88 college students, dividing them into “good” and “poor” planners and eventually describing the main difference between the two groups being their perception and description of planning, rather than the proportion of completed goals [20]. Adopting realistic and reasonable daily plans for tasks is linked to short-term results, which positively reflects a sense of accomplishment. gathering small achievements over a short period of time is accompanied by greater productivity in the long term. Regarding college students, the matter is also related to gathering information over a short period and then reviewing it later in a gradual and sequential manner, what is known as “spacing effect”, which simply states that studying is more effective when spacing out study sessions as “repetitions” [21]. It was suggested by another study that spacing is better than mass learning when students delay their studies until near the exam, a suggestion that may explain the relationship between adapting daily organization of time and the higher GPA [22]. About half (52.1%) of study participants agreed that they tend to delay/postpone their tasks which is related to mass learning described earlier, although not found to be statistically associated with student’s GPA, especially when a large percentage (42.9%) of participating students agreed that they can manage workload effectively.

Students’ ability to adapt and be flexible when changes of plans occur was associated with higher GPA (Table 3). The positive relationship between flexibility and GPA was also mentioned by another study [23]. Nevertheless, it was mentioned in other articles that while achieving high GPA through flexibility and adaptability, those students experienced a tremendous psychological challenges that lead eventually to improper mental health [24].

A recent 2024 study explained another relationship, as flexibility is the ability to still produce effectively when changes in plans occurs, on the other side when the student is unable to adapt and manage time, leading to procrastination and subsequently negatively affecting psychological wellbeing [25]. It is important to put in mind the many other factors affecting academic performance and psychological wellbeing. The proposed studies about flexibility and the conflicting ideas about whether it is related negatively or positively to psychological health needs another specific study addressing this issue in Iraq.

In our study students’ GPA was found to have a statistically significant association with insufficient sleeping pattern that lead to a feeling of laziness, and ruined studying mood among students. Insufficient quantity (sleep deprivation) has numerous effects upon academic performance and achievement. A study stated that sleep deprivation was associated with daytime sleepiness and consequently associated with lower GPA among college students alongside poor academic performance, on the other hand, students that has long sleeping hours (> 9 h) had higher GPAs than those with short sleeping hours (< 6 h) [26] furthermore, a cross-sectional study by Jalali et al., found no significant association between sleep quality and academic achievement [27].

In simple terms, sleep deprivation has a number of effects on academic performance and achievement. Physiological effect related to circadian rhythm, psychological effect, health-related effects related to the number of behaviors adapted by poor sleepers in order to cope with academic and daily life demands [28, 29]. It is important to set up a daily routine that maintains a wake-sleep cycles sufficient to establish a natural circadian response, in order to be fully energized and engaged into college activities and academic requirements and dealing with information and memorization in the best possible way. It was found that disruption of circadian rhythm leads to irregularity in cortisol secretion and negative impact on cognitive function and has an association with depression [30]. A recent study by Aldabbour et al., explored with relationship between mental health and sleep among medical students, The study showed that lack of sleep among them was linked to the emergence of symptoms of depression, anxiety, stress, and general feeling of life dissatisfaction, a finding that supports our study results [31].

Of the study limitations, as it is a cross sectional study, the temporality and cause-effect cannot be precisely determined. In addition, aspects of students’ psychological well-being (mood and socioeconomic status) were not given enough weight, nevertheless, gathering information about these factors is rather an exquisite process that needs to be analyzed precisely using a specific questionnaire.

The strength of our study lies in adequate sample size, allowing generalization of the results. Secondly, choosing the study to be conducted on medical students specifically gives a reliable reflection of the effect of time management on academic achievement as medical college is a demanding discipline, and studying medicine requires students to organize their time effectively in order to achieve high grades.

## Conclusions

Both time management and academic performance were modest among students. Daily time management and adequate sleep were significantly associated with higher GPA. Focused students’ education and comprehensive strategies to enhance effective time management skills among them are recommended.

## Data Availability

The data are available from the corresponding author on request.

## Abbreviations

GPA: Grade Point Average
OED: Oxford English Dictionary
SPSS: Statistical Package for the Social Sciences
